# Evaluation of liver function and electroacupuncture efficacy of animals with alcoholic liver injury by the novel imaging methods

**DOI:** 10.1038/srep30119

**Published:** 2016-07-22

**Authors:** Dong Zhang, Xiao-jing Song, Shun-yue Li, Shu-you Wang, Bing-jun Chen, Xiao-Dong Bai, Li-mei Tang

**Affiliations:** 1 Department of biomedical engineering, Institute of Acupuncture & Moxibustion, China Academy of Chinese Medical Sciences, 100700, Beijing, China

## Abstract

Imaging methods to evaluate hepatic microcirculation (HM) and liver function (LF) by directly monitoring overall liver tissue remain lacking. This study establish imaging methods for LF that combines Laser speckle perfusion imaging (LSPI) and *in vivo* optical imaging (IVOI) technologies to investigate changes of hepatic microcirculation and reserve function in the animals gavaged with 50% ethanol (15 ml/kg·bw) for a model of acute alcoholic liver injury (ALI), and for evaluation of electroacupuncture (EA) effect. The liver blood perfusion and indocyanine green (ICG) distribution were observe by LSPI and IVOI separately. After EA, the livers were collected to measure the levels of alanine aminotransferase (ALT), aspartate aminotransferase (AST), thromboxane A (TXA_2_), prostacyclin (PGI_2_) and endothelin (ET). The acquisitions of newly established LSPI of liver and ICG *in vivo* fluorescence imaging (ICG-IVFI), combining the results of other indexes showed: hepatic microcirculation perfusion (HMP) significantly reduced, ICG metabolism reduced, and ALT/AST increased in animal model with acute ALI. EA can reverse these changes. The use of LSPI of liver and ICG-IVFI, which was novel imaging methods for LF established in this study, could display the LF characteristics of ALI and the EA efficacy.

Alcoholic liver injury (ALI) is also known as alcoholic liver disease (ALD), and the proportion of these patients among all patients with liver disease has been steadily increasing^1^. Early alcoholism can cause mild ALI, and sustained alcoholism can cause alcoholic fatty liver, alcoholic hepatitis, alcoholic liver fibrosis, and alcoholic liver cirrhosis^2^. In some areas, the proportion of people with mild ALI in ALD was second only to that of people with alcoholic hepatitis^3^ causing an increasing health risk. Studies have shown that after the intake of high concentrations of ethanol, excessive acetaldehyde causes the release of aldehyde dehydrogenase, hepatocyte hypoxia and oedema, the destruction of hepatocytes and hepatic sinusoidal structure, the compression of blood vessels in the hepatic microcirculatory system, and blood circulation disorders^4^,^5^. Additionally, ethanol can directly induce portal contraction, the reduction of hepatic circulation, microcirculation disorders, and the aggravation of liver injury. Therefore, alcoholic liver injury and microcirculation are very closely related^6^.

As an alternative medicine therapy, acupuncture has been accepted by a certain population in many countries because it is simple and can cure many diseases^7^. In 1997, the National Institutes of Health (NIH) of the United States recognized the efficacy of acupuncture for some diseases^8^. In 2002, the World Health Organization (WHO) released a review and an analysis of clinical acupuncture trials, and the efficacy of acupuncture for more diseases is being studied^9^. Currently, acupuncture is applied in the treatment of various liver diseases^10^. The clinical application of acupuncture is widely studied for its use in the treatment of alcohol dependence and alcoholism^11^,^12^,^13^. However, the study of acupuncture treatment for use in treating alcohol-induced liver disease remains relatively rare.

Laser speckle perfusion imaging (LSPI) is an emerging perfusion imaging technology that combines the advantages of high image density (500 × 700 pixels) and high imaging speed (60 frames/sec), thus enabling the accurate capture of dynamic microcirculation changes^14^; this technique achieves good results in the detection of perfusion in organs and tissues with rich microcirculation systems, such as the brain and spinal cord^15^,^16^,^17^. *In vivo* optical imaging (IVOI) is a recently developed optical imaging technology that combines the use of bioluminescence and fluorescence imaging. This technology mainly uses chemical bioluminescence and luciferase luminescence to label organs, tissues, and cells at different levels *in vivo*, thereby determining changes in body function activities by detecting the distribution of and changes in fluorescence^18^,^19^. This technique is characterized as an intuitive, non-invasive, rapid and sensitive imaging method that can directly measure the area and intensity of luminescence in a live animal to investigate cellular metabolic activity and genetic behavior. This technique is an effective new technology for studying functional activity in a whole body and in certain organs of an organism, gene expression patterns and markers, cell tracking, and interactions between proteins^20^,^21^. Currently, this technology is mostly applied to *in vivo* observation for drug targeting^22^, organ transplantation monitoring^23^, the development of disease models^24^, and the treatment of viral infection^25^,^26^. However, the application of both of these imaging techniques to monitor liver function has rarely been reported, and its application to the study of electroacupuncture (EA) efficacy is even rarer.

In current clinical diagnostics and experimental research, methods for imaging the function of liver with ALI, especially mild and moderate injury, are lacking, consequently affecting our understanding of the early pathological changes that occur in this disease. Few simple and easy therapeutic interventions are available to treat the early stages of this disease, leading to exacerbation in patients with mild disease. This study applied LSPI and IVOI technologies to assess ALI for the first time and to learn more about the pathological process of this form of injury. Moreover, EA stimulation was applied to an animal model of ALI to explore the possibility of EA treatment for this disease.

## Results

### Liver morphology of animals with acute ALI

**Figure 1:**
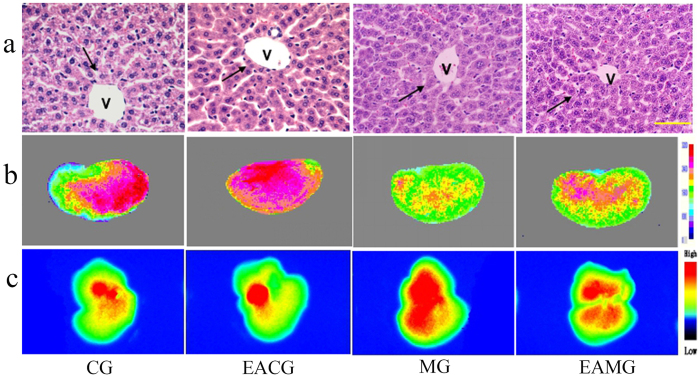
Visualization of Morphology, Laser speckle perfusion imaging (LSPI) and ICG-*in vivo* fluorescence imaging (ICG-IVFI) of liver tissues. (**a**) HE staining of liver tissue (×400); scale bar: 10 μm, V indicates vascular tissue, and the black arrows show liver cells. In the CG and EACG, the liver tissue structure was normal. In the MG and EAMG, the cells were swollen; the liver cell nuclei were pushed to one side; the hepatic sinusoid was under pressure; the hepatic cord was disordered; and the lobular structure was unclear. (**b**) LSPI images of the liver. In the CG, the microcirculation perfusion was rich. In the EACG, the microcirculation perfusion was the most rich. In the MG, the microcirculation perfusion was low. In the EAMG, the microcirculation perfusion after EA was increased compared to the level without EA. (**c**) ICG-IVFI of liver lobe at 90 min after ICG injection. The ICG FI from high to low was MG, EAMG, CG and EACG.

HE staining of liver tissue in the control group (CG) showed the following results: liver cells of similar size, rounded nuclei with clear nucleoli, pink-stained cytoplasm, no infiltration of inflammatory cells in the portal area, orderly arrangement of the hepatic cord in a radial pattern, and normal lobular structure (Fig. 1a). The morphological characteristics of the liver tissue in the EA control group (EACG) were the similar as CG’ (Fig. 1a). The morphology of the liver tissue in the model group (MG) showed significant changes: the liver cells were swollen and exhibited increased volume, the nuclei of the liver cells were pushed to one side, especially those in liver cells at the outer periphery of the lobule, the hepatic sinusoid was under pressure, the hepatic cord was disordered, and the lobular structure was unclear (Fig. 1a). The structure of the liver tissue of the EA model group (EAMG) exhibited damage; compared with the MG, there was not obvious difference (Fig. 1a).

### Hepatic microcirculation perfusion (HMP) and changes after EA

**Figure 2:**
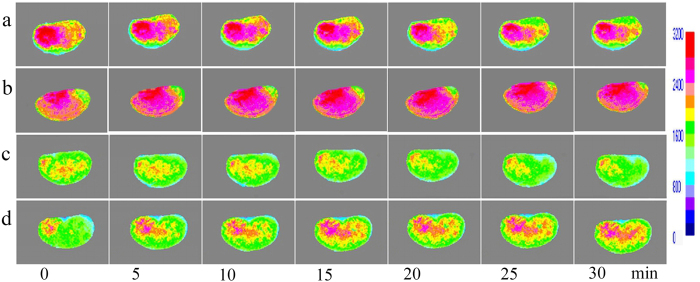
Visualization of Laser speckle perfusion imaging (LSPI) for the liver. (**a**) Hepatic LSPI images of CG at each time point; (**b**) Hepatic LSPI images of EACG at each time point; (**c**) Hepatic LSPI images of MG at each time point; (**d**) Hepatic LSPI images of EAMG at each time point (0 min: before EA, 5–30 min: 5–30 min after EA), showing the gradually increase in perfusion after EA.

**Figure 3:**
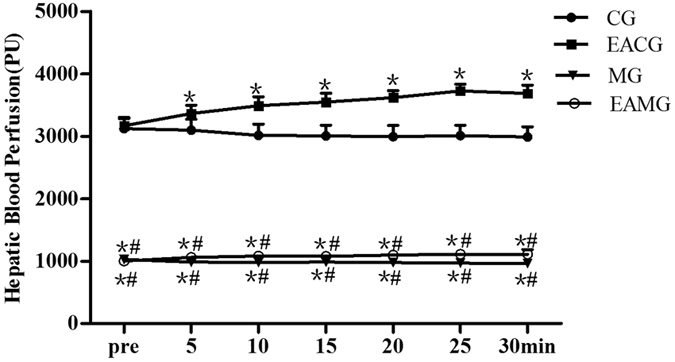
Quantifications of HMP data among groups. HMP time plotted against perfusion amount. *P < 0.05, vs CG. ^#^P < 0.05, vs EACG (one-way ANOVA with LSD’s two groups comparison test). Data are mean ± SD.

The HMP in the CG was rich, with little change during the 30 min of continuous observation (Fig. 2a). After EA stimulation, HMP of EACG was gradually increased (Fig. 2b). The HMP of MG was low, with no significant change during the 30 min of continuous observation, and perfusion was maintained at a low level (Fig. 2c). The HMP of AG distribution characteristics in the EAMG before EA were similar to those in the MG, showing low perfusion. After EA stimulation, the HMP in the EAMG was slowly increasing (Fig. 2d). The HMP in the CG and EACG was more than three times that in the MG and EAMG. After EA 5 min, the HMP of EACG was higher than that in the CG. At each time point, the HMP in the EACG and CG both were significantly higher than that in the MG and EAMG (P < 0.05); but the perfusion of the EAMG after EA was higher than that of the MG (Fig. 3).

### ICG-*in vivo* fluorescence imaging (ICG-IVFI) of liver and the effect of EA

**Figure 4:**
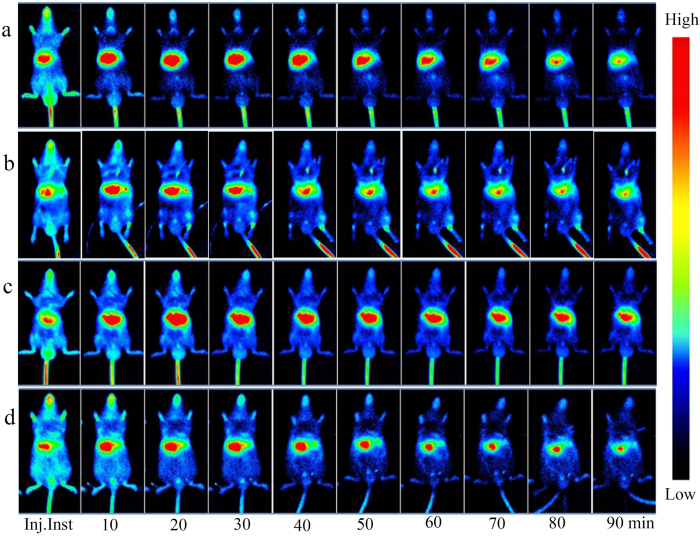
Visualization of ICG-*in vivo* fluorescence imaging (ICG-IVFI) for the liver. (**a**) ICG-IVFI images of CG. Immediately after the ICG injection, sluggish aggregation of fluorescence distribution in liver was observed; at 10–30 min, the FI was gradually increased; at 20–30 min, the FI reached the highest level, and the fluorescence area was the largest; at 30–40 min, the FI was slowly decreased, and the fluorescence area was reduced; at 60–90 min, the FI was significantly reduced. (**b**) ICG-IVFI images of EACG. The 10-30 min indicates the EA time, whereas 40–90 min corresponds to the 10–60 min after EA. At each time point, the fluorescence distribution characteristic of EACG was the same as CG’; but the FI was lower than CG’ after EA. (**c**) ICG-IVFI images of MG. The fluorescence intensity was significantly higher than that of the control group; at 40–90 min, the fluorescence intensity remained at a high level. (**d**) ICG-IVFI images of EAMG. The 10–30 min indicates the EA time, whereas 40–90 min corresponds to the 10–60 min after EA. Within 30 minutes of EA, the FI reached a maximum; 10–60 minutes after EA, the FI was gradually decreased, and the FI and area were intermediate between those of the CG and the MG.

**Figure 5:**
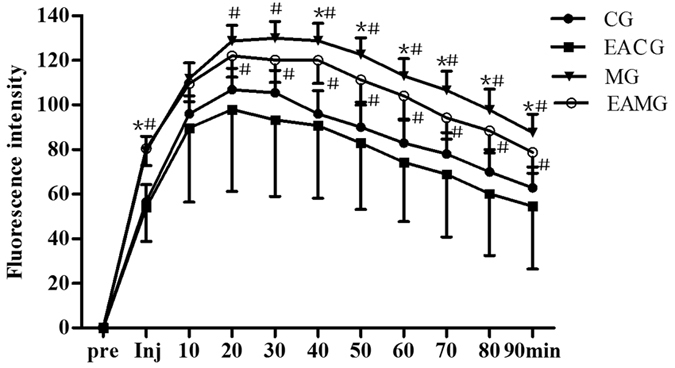
Quantifications of ICG-FI data among groups. Liver ICG time plotted against intensity. *P < 0.05, vs CG. ^#^P < 0.05, vs EACG (one-way ANOVA with LSD’s two groups comparison test). Data are 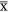 ± SE.

The results of IVOI showed that immediately after the ICG injection, sluggish aggregation of fluorescence distribution was observed in the livers of the animals in all groups; at 10–30 min after injection, the fluorescence intensity (FI) was gradually increased; at 20–30 min after injection, the FI reached the highest level, and the fluorescence area was the largest (Fig. 4). For the CG, at 30 min after injection, the FI was slightly decreased, and the fluorescence area was reduced; at 60 min after injection, the FI was significantly reduced (Fig. 4a). Except for FI, the characteristic of ICG fluorescence distribution in other groups was the similar to CG’ at every time point. At each time point, the FI of EACG was lower than that of CG. The ICG FI of the MG was higher than that of other groups (Fig. 4b–d); immediately and 40–90 min after the injection, the differences in the ICG FI between the MG and the CG were statistically significant (P < 0.05). At 20–90 min after the injection, the differences in the ICG FI between MG and EACG, between EAMG and EACG were statistically significant (P < 0.05) (Fig. 5). Liver lobes were obtained from two mice in each group at 90 min after ICG injection for individual imaging; the results showed that the ICG FI of the liver tissue from high to low was MG, EAMG, CG and EACG (Fig. 1c). It could be seen from the results that the FI characteristic of liver tissue in each group was the same as the HMP characteristic of each group (Fig. 1b,c).

### Vasoactive substance and transaminase contents in livers of each group

**Figure 6:**
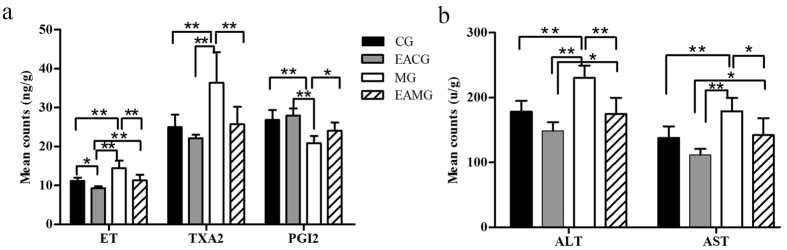
Quantifications of hepatic vascular regulator and transaminase data among groups. (**a**) Comparison of vascular regulator level. *P < 0.05, **P < 0.01 (one-way ANOVA with LSD’s two groups comparison test). Data are mean ± SD. (**b**) Comparison of transaminase level. *P < 0.05, **P < 0.01 (one-way ANOVA with LSD’s two groups comparison test). Data are mean ± SD.

The level of the vasodilator PGI_2_ in the liver of animals in the EACG was highest (27.92 ± 1.9 ng/g), followed by the CG (26.82 ± 2.58 ng/g), EAMG (24.06 ± 2.12 ng/g) and the MG (20.85 ± 1.88 ng/g). The level of PGI_2_ in the MG was significantly lower than those of other groups (P < 0.05 and P < 0.01) (Fig. 6a).

The levels of the vasoconstrictors ET and TXA_2_ were lowest in the liver of the animals in the EACG (9.33 ± 0.49 and 22.14 ± 0.89 ng/g); the third in the CG (11.20 ± 0.92 and 24.96 ± 3.27 ng/g); the second in the EAMG (11.35 ± 1.42 and 25.77 ± 4.41 ng/g); and the highest in the MG (14.45 ± 1.15 and 36.36 ± 7.91 ng/g). A comparison of the levels of ET and TXA_2_ among the various groups showed that the levels of the MG were significantly higher than those of other groups (P < 0.01). The levels of ET in the EAMG was significantly higher than that in the EACG (P < 0.01) (Fig. 6a).

The level of ALT in the liver of the animals was the lowest in the EACG (156.71 ± 35.99 U/g), the third in the EAMG (174.52 ± 25.13 U/g), the second in the CG (178.26 ± 16.78 U/g), the highest in the MG (230.26 ± 18.99 U/g). The level of AST was the lowest in the EACG (116.33 ± 26.69 U/g), the third in the CG (137.82 ± 17.85 U/g), the second in the EAMG (142.12 ± 26.22 U/g), the highest in the MG (178.82 ± 20.63 U/g). The levels of ALT and AST in the liver of the animals in the MG were significantly higher than those of other groups (P < 0.05 and P < 0.01). The levels of ALT and AST in the liver of EAMG were significantly higher than those of EACG (Fig. 6b).

## Discussion

Liver is involved in bodily secretion, excretion, and biotransformation and is a vital organ for the synthesis of many cytokines with various physiological functions. In ALI, various diseases are overlapped to a considerable degree; currently, the indicators and corresponding pathological changes of ALI remain controversial. Hepatic microcirculation (HM) is an important element of liver metabolism and signal transduction, and changes in HMP are closely related to liver function^27^. Therefore, alcohol damage is likely to be accompanied with blood circulation disorders. Understanding changes of HM in ALI is very significant for the study of the occurrence, development and treatment of this disease. Previously, direct imaging methods have monitored HMP mainly through the evaluation of blood flow in large and medium blood vessels (e.g., the hepatic portal vein and hepatic artery) to reveal HMP. Imaging methods to evaluate HMP by directly monitoring the overall HM remain lacking. Our laboratory applied laser Doppler blood flow imaging and LSPI technology for the first time to display perfusion over the entire surface of an animal stomach^28^. On this basis, this study established a liver LSPI method for the intuitive and dynamic observation of the HMP in livers with alcoholic injury.

The results of our experiments showed that damage to rat liver tissue was observed 12 hours after oral administration with 50% ethanol, including swollen liver cells, hepatic sinusoids under pressure, unclear liver cords and lobular structure, damaged liver cells, and increased levels of AST and ALT. The results of LSPI for liver showed that HMP in the MG was significantly decreased to only 20% of the level of the CG; this is one of the main results obtained by the imaging method established in this study. In the early stage of acute ALI, the cells are swollen and the hepatic sinusoid is under pressure, and the structure of the lobule is disordered, leading to decreased HMP and the aggravation of liver lesions. The method of liver LSPI we established successfully demonstrated the effect of EA on HMP increase in healthy animals and ALI model animals, providing a new method for the detection and study of HMP.

Similar to other parts of the body, blood perfusion in liver is regulated by vasoactive substances. When ALI occurs, levels of the vasoconstrictor ET and TXA_2_ in liver tissue were increased, and levels of the vasodilator PGI_2_ were decreased. After EA stimulation, the levels of the above substances of animals with acute ALI were partially restored. It was also observed that HMP was significantly decreased. EA stimulation changed the levels of vascular regulators in the liver tissue to promote HMP increases. Meanwhile, the levels of AST/ALT also were decreased. This perfusion improvement could provided the beneficial conditions to restore the liver cell damage after acute ALI. It was considered that the self-regulation effect of body caused by EA stimulation could be relative to body state. In this study, it shown that the EA effect on HMP increase in healthy animals was stronger than that in ALI model animals. This result was consistent with our previous studies^29^,^30^.

The ICG clearance assay is most commonly used to evaluate liver function (LF). The rate of ICG clearance is positively correlated with the effective blood flow in liver^31^,^32^,^33^. After ICG is intravenously injected into the body, it can immediately bind to plasma proteins and is therefore rapidly distributed throughout the body in the blood circulation and is specifically taken up by liver cells. After being cleared by the hepatocytes in the liver, ICG is finally excreted in a free form with the faeces^34^,^35^. At approximately 20 min after ICG injection, the blood ICG reaches maximum concentration; thereafter, the concentration gradually declines due to liver clearance at a linear rate. When a disease associated with a lower effective blood flow and a lower number of liver cells occurs in the liver, the clearance rate of plasma ICG is significantly decreased, and ICG s retained in the liver tissue. Therefore, the rate of ICG clearance could reflect the LF^36^,^37^. Because ICG is cleared in the liver, this study combined IVOI technology with the classic ICG excretion assay. By comparing the ICG’ FI and the fluorescence distribution area in liver at different time points, the retention time of ICG in the liver with ALI was obtained to investigate the time-effect relationship for EA with the retained amount of ICG in the liver. The results of ICG-IVFI showed that after intravenous injection of ICG in the animals, the ICG in liver was gradually increased; the ICG’ FI was greatest at 20–30 min after injection, and the distribution area was the largest; after this time, the FI and the area were slowly decreased. This finding is fully consistent with the result of a classical ICG clearance assay. However, this study noninvasively observed the dynamic ICG distribution, which more intuitively reflects the characteristic of liver ICG clearance. This study also found that the ICG’ FI and the retention time in the animal’ liver with ALI were significantly higher than and longer than those of the healthy animals, indicating that liver metabolism was impaired even in the early stage of ALI, resulting in a slow IGC clearance rate. With EA stimulation, the ICG’ FI in liver was lowest in healthy mice; the ICG’ FI in EAMG was lower than that in the MG, and EA shortened the ICG retention time in the damaged liver, indicating that EA promoted the metabolism of ICG in the liver. This is another major result obtained by the method of hepatic ICG-IVFI established in this study. We believe that if hepatic LSPI combined with ICG-IVFI, the new imaging technologies will become meaningful experimental means for studying LF and acupuncture efficacy.

Based on the experimental results, our analysis showed that EA stimulation could activate the endogenous microcirculation regulatory system in alcoholic liver injury. In addition, EA stimulation promoted vascular endothelial cells in liver tissues to express a large amount of eNOS to increase the synthesis and release of NO, cause vasodilation, and increase blood flow^38^. When the amount of blood perfusion is increased, the blood flow increases the friction on blood vessel walls. After vascular endothelial cells and nerves sense the changes in blood vessel wall friction, the biological activities change^39^ to increase the release of the vasoactive substance PGI_2_ and decrease the release of the vasoconstrictors ET and TXA_2_, thereby promoting vasodilation and increasing blood flow perfusion in the liver. The improvement in liver microcirculation could, to some extent, promote the repair of injured hepatocytes. After EA, AST and ALT levels in the livers of the alcoholic liver injury animals improved, ICG excretion in the liver accelerated, and liver function improved.

Currently, evaluation methods for the detection of hepatic blood flow include Doppler ultrasound, X-ray CT angiography, digital subtraction angiography, MRI, and PET imaging techniques. However, these methods can only detect the structure or blood flow values of large and medium blood vessels in organs. There are still no good imaging methods to detect microcirculation in organs. The ICG clearance test was used to evaluate hepatocytes damage and LF by measuring the ICG level of serum. There are not any methods to measure the ICG level of liver tissue directly. The novel methods of hepatic LSPI and ICG-IVFI established in this study had displayed the characteristic of hepatic microcirculation and ICG clearance and the EA effect in physiological and pathological condition. The results indicate that the combination of hepatic LSPI and ICG-IVFI techniques used in the measurement, diagnosis and evaluation of LF would have actual meaning and application foregrounds.

In conclusion, in animals with acute ALI, we found that liver structure was damaged, HMP was decreased, LF was impaired, and ICG clearance of liver was slowed. EA stimulation altered the levels of vasoactive substances to increase HMP and promote ICG clearance, thereby improving HM and reserve function, protecting LF in animals with acute ALI. The novel methods of hepatic LSPI and ICG-IVFI established in this study have practical value for the intuitive demonstration of LF changes. Otherwise, that EA stimulation had some effects on the LF in the animal with acute ALI also was observed. It prompt that EA intervention will became quite meaningful in the research of prevention and treatment of ALI because it is a nontoxic therapy.

## Methods

### Experimental animals and grouping

The experimental animals used in this study included 180 healthy adult Kunming mice with an average age of 3–4 weeks, weights of 20–22 g, and a male/female ratio of approximately 1. The study also used 40 healthy adult male Sprague-Dawley (SD) rats with weights of 160–180 g. All animals were provided by the Experimental Animal Center of the Chinese Academy of Military Medical Science (license: SCXK-(Army) 2011-004). They were treated in accordance with international standards for the rearing and use of laboratory animals, and the entire experiment was performed in accordance with the protocol approved by the Animal Ethics Committee of the Institute of Acupuncture and Moxibustion at the China Academy of Chinese Medical Science (No.20140013). The animals were divided into CG, EACG, MG and EAMG. After being fasted for four hours, the animals in the MG and EAMG were weighed and then orally gavaged with 50% ethanol (15 ml/kg·bw) once to establish the ALI model. The animals in the CG and EACG were gavaged with normal saline (15 ml/kg·bw). All animals were continuously reared for 12 hours after the gavage, and then other indicators were observed. The EA was performed in EACG and EAMG as follows: an 0.18 × 13 mm acupuncture needle was used to pierce the bilateral “Zusanli” (ST36) at the depth of 3 mm. The acupuncture needle was connected to an electro-acupuncture device (MBT-1 synchronization pulse therapeutic apparatus linked with a microcomputer, Zhejiang Huayin Electronics Co., Ltd., China). The EA parameters were as follows: continuous wave voltage of 4–6 V at a frequency of 1 Hz and EA duration of 30 min. The same oral administration with 50% ethanol and EA methods were applied to the mice and the rats.

### Observation of liver tissue morphology and hepatic microcirculation perfusion

(*HMP*) by LSPI One hundred mice were randomly divided into the CG, the EACG, MG and the EAMG (25 mice in each group). At 24 h after oral administration with 50% ethanol, an intraperitoneal injection of 2% sodium pentobarbital (2.5 ml/Kg) was conducted. After the mice were fully anesthetized, the abdominal cavity was cut open along the abdominal midline below the xiphoid, and the liver was dissociated and placed *in vivo* on the abdominal wall. The animals were placed in an incubator at 30 ± 0.5 °C under 80–90% humidity. HMP was detected using a Moor-FLPI perfusion imager (Moor Co., UK. Scanning area: 3 × 2 cm; pixel resolution: 576 × 768; image acquisition rate: 25 frames/sec; 10-frame continuous acquisition mode - time interval: 1 s and exposure time: 20 ms). Liver speckle perfusion (LSP) images were acquired every 5 min; 7 images were acquired in 0–30 min. For the mice in the EACG and EAMG, LSP images of the liver before EA and within 30 minutes of the EA were obtained. The LSP images obtained were analyzed using the moorFLPI-V2.0 system software, and the average HMP of the selected area at each time point was retrieved (PU represents the perfusion quantitative unit recorded within the Moor-FLPI system).

After the LSP imaging detection, five mice were randomly killed in each group. A piece of liver tissue (1 × 0.5 × 0.2 cm) was collected at 5 mm from the edge of the liver. After fixing with 10% formalin, paraffin sections were prepared at a thickness of 8 μm, followed by conventional HE staining. The structural and pathological changes of the liver tissue and cells were observed under an optical microscope.

### ICG *in vivo* fluorescence imaging (ICG-IVFI) of liver

Eighty mice were randomly divided into the CG, EACG, MG and EAMG (20 mice in each group). At 24 h after oral administration with 50% ethanol, the mice were subjected to an intraperitoneal injection of 2% sodium pentobarbital (2.5 ml/Kg). After the mice were fully anesthetized, 10 μg/mL of ICG solution (Suizhou Jiake Pharmaceutical and Chemical Industry Co., Ltd. China) was injected through the tail vein (12.5 μl/g BW). The mice were then flattened in a prone position in the recording dark chamber of an FX PRO multi-modal *in vivo* imaging system (Carestream Health Inc., USA). The instrumental parameters were set as follows: excitation wavelength, 790 nm; emission wavelength, 830 nm; exposure time, 10 seconds. Images were acquired immediately after the ICG injection; *in vivo* fluorescence images were then collected at 10 time points every 10 min for 0–90 min. For the mice in the EACG and EAMG, EA was performed immediately after the ICG injection; the images of ICG-IVFI were acquired at 10 time points at 10, 20 and 30 min during EA and at 10, 20, 30, 40, 50 and 60 min after EA. The fluorescence intensity (FI) during the ICG cleaning process in the liver was analyzed.

Two mice were randomly killed in each group 90 min after the ICG injection. The entire liver lobe was immediately removed by laparotomy and placed in the recording dark chamber of the *in vivo* imaging system to acquire fluorescence images of the liver lobe using the same instrumental parameters as those described above.

### Detection of transaminases and vascular regulators in liver

Thirty rats were randomly divided into the CG, EACG, MG and the EAMG (10 mice in each group). The mice were anaesthetised using an intraperitoneal injection of 2% sodium pentobarbital (2.5 ml/Kg). EA stimulation was then applied to the rats in the EACG and EAMG for 30 minutes; EA was not applied to the rats in the CG and MG. After 30 minutes, all rats were euthanised.

Determination of liver transaminase content: Liver tissue (0.5 g) was homogenized and centrifuged in a high-speed centrifuge, and the supernatant was obtained. The contents of alanine aminotransferase (ALT) and aspartate aminotransferase (AST) were detected using the method of Lai (ALT and AST assay kits were obtained from Nanjing Jiancheng Bioengineering Institute and Beijing Kangrun Yueze Technology Co., Ltd.), and the results were recorded and analyzed using an UV-visible spectrophotometer (U-5100; Hitachi Inc., Japan) at 190–900 nm; the spectral bandwidth was adjusted to 0.1–5 nm.

Determination of liver vascular regulator contents: Liver tissue (0.5 g) was homogenized in 0.9% NaCl solution (2 μl/μg) and centrifuged in a high-speed centrifuge, and the homogenate was centrifuged for 3000 rpm at 4 °C for 10 min to obtain the supernatant. The thromboxane A (TXA_2_), the prostacyclin (PGI_2_) and the endothelin (ET) were respectively determined using an TXA_2_ ELISA kit (Beijing Sinouk Institute of Biological Technology, Beijing, China), PGI_2_ ELISA kit (Beijing Sinouk Institute of Biological Technology, Beijing, China), an ET assay kit (Beijing Sinouk Institute of Biological Technology, Beijing, China) following the manufacturer’s protocol. A STAT FAX 2100 automatic microplate reader (Awareness Technology Inc., USA) was used for detection and analysis.

### Statistical analysis

The means of perfusion and the average intensity of ICG fluorescence in the animal livers in each group were calculated at each time point to investigate the changes in the parameters with time. The means of ALT, AST, TXA_2_, PGI_2_ and ET were calculated for each group. One-way ANOVA with a LSD test was used to test differences in each indicator between groups using SPSS 19.0 statistical software. The data were represented as means ± SD and 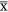 ± SE. Differences with P < 0.05 were considered statistically significant.

## Additional Information

**How to cite this article**: Zhang, D. *et al*. Evaluation of liver function and electroacupuncture efficacy of animals with alcoholic liver injury by the novel imaging methods. *Sci. Rep.* **6**, 30119; doi: 10.1038/srep30119 (2016).
